# *UnitCell Tools*, a package to determine unit-cell parameters from a single electron diffraction pattern

**DOI:** 10.1107/S2052252521007867

**Published:** 2021-08-20

**Authors:** Hong-Long Shi, Zi-An Li

**Affiliations:** a School of Science, Minzu University, 27 Zhong guancun South Avenue, Haidian District, Beijing 100081, People’s Republic of China; b Institute of Physics, The Chinese Academy of Sciences, No. 8, 3rd South Street, Zhongguancun, Haidian District, Beijing 100190, People’s Republic of China

**Keywords:** unit cell, Bravais lattice, electron diffraction, *SAED*, *HOLZ*

## Abstract

Electron diffraction techniques in transmission electron microscopy (TEM) have been successfully employed for determining the unit-cell parameters of crystal phases, albeit they exhibit a limited accuracy compared with X-ray or neutron diffraction, and they often involve a tedious measurement procedure. Here, a new package for determining unit-cell parameters from a single electron diffraction pattern has been developed. The essence of the package is to reconstruct a 3D reciprocal primitive cell from a single electron diffraction pattern containing both zero-order Laue zone and high-order Laue zone reflections. Subsequently, the primitive cell can be reduced to the Niggli cell which, in turn, can be converted into the unit cell. Using both simulated and experimental patterns, we detail the working procedure and address some effects of experimental conditions (diffraction distortions, misorientation of the zone axis and the use of high-index zone axis) on the robustness and accuracy of the software developed. The feasibility of unit-cell determination of the TiO_2_ nanorod using this package is also demonstrated. Should the parallel-beam, nano-beam and convergent-beam modes of the TEM be used flexibly, the software can determine unit-cell parameters of unknown-structure crystallites (typically >50 nm).

## Abstract 2

The *UnitCell Tools* package was developed to determine unit-cell parameters of a crystal from a single electron diffraction pattern with high-order Laue zone reflections acquired in the conventional transmission electron microscope.

## 1. Introduction   

Unit-cell parameters and symmetry elements are two fundamental crystallographic quantities for characterizing a crystal structure. In a structural study, accurate measurement of unit-cell parameters for a crystalline phase is considered to be the first step towards identifying a crystalline phase with known structures (Pecharsky & Zavalij, 2003 ▸; Williams & Carter, 2009 ▸; Young, 1995 ▸) or solving the crystal structure of unknown crystals (Le Bail *et al.*, 1988 ▸; David *et al.*, 2006 ▸). X-ray and neutron diffraction are two well established methods for the unit-cell determination of single-phase materials. However, their broad irradiation beam makes them ill-suited for probing finite-sized samples (Baer *et al.*, 2008 ▸), especially those in the form of nanoscale materials (Shi, Zou *et al.*, 2019 ▸), inclusions or precipitates in alloys (Antion *et al.*, 2003 ▸), and structurally modulated materials that are multiphase such as multilayers and superlattices (Collier *et al.*, 1998 ▸). By contrast, high-energy electron diffraction in transmission electron microscopy (TEM) can probe finite-sized crystals, though the measurement precision of unit-cell parameters is relatively poor for several practical reasons (Mugnaioli *et al.*, 2009 ▸; Capitani *et al.*, 2006 ▸; Hou & Li, 2008 ▸).

Several approaches of electron diffraction in TEM for the unit-cell determination of a crystal have been developed. The first employs (1) the search–match method (Lábár, 2008 ▸; Shi, Luo & Wang, 2019 ▸; Altomare *et al.*, 2019 ▸; Zuo *et al.*, 2018 ▸), which involves measuring *d*-spacings of reflections in a powder electron diffraction pattern and comparing *d*-spacings with those of the known structures in crystal databases; and (2) the pattern-indexing method (Williams & Carter, 2009 ▸; Shi, Luo & Wang, 2019 ▸),which involves measuring *d*-spacings and interplanar angles in a zone-axis diffraction pattern and performing the pattern-indexing procedure based on the known structures. However, this approach cannot be used in the case of unknown structures. The second approach is to construct a 3D reciprocal primitive cell based on zone-axis electron diffraction patterns: (1) the multi-pattern method (Zou *et al.*, 2004 ▸) requires a tilt series of zone-axis patterns (at least three) to reconstruct 3D reciprocal space using the geometric relationship of patterns; (2) the two-pattern method (Li, 2019 ▸; Zhao *et al.*, 2008 ▸) requires two zone-axis patterns and their interzonal angles to determine unit-cell parameters; and (3) the one-pattern method is to construct a 3D reciprocal primitive cell by making use of structural information present in the convergent-beam electron diffraction patterns (Ayer, 1989 ▸; Zuo, 1992 ▸, 1993 ▸). Some excellent electron diffraction software has been developed to determine unit-cell parameters, *e.g.* *TRICE* (Zou *et al.*, 2004 ▸), *TEMUC3* (Li, 2019 ▸) and *QtUCP* (Zhao *et al.*, 2008 ▸) *etc*. These analysis packages require at least two zone-axis patterns recorded by a tilt series; however, this limits the application of the nanosized crystallite and the electron-sensitive materials for the time-consuming crystal tilting process.

In this work, we re-examine the geometrical description of the zone-axis electron diffraction and develop the package ‘*UnitCell Tools*’ to determine unit-cell parameters from a single electron diffraction pattern with both ZOLZ and HOLZ reflections. We describe the working principles of the software and demonstrate the processes of constructing the 3D primitive reciprocal cell, converting the primitive reciprocal cell to the Niggli reduced cell and then to the conventional unit cell using both simulated electron diffraction and experimental data. We also address the experimental aspects (diffraction distortion, zone-axis misorientation and the use of high-index or arbitrary zone-axis pattern) on the robustness and accuracy of the proposed software. Moreover, we experimentally test the applicability of the software to determine the unit-cell parameters of nanocrystallites using diffraction patterns from both CBED and nano-beam electron diffraction (NBED) methods.

## 2. Fundamentals of the method   

**Figure 1:**
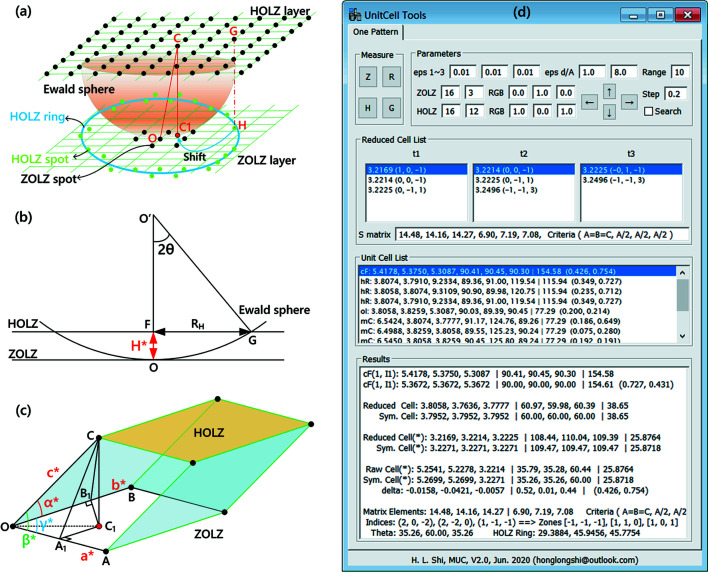
Determination of unit-cell parameters from an electron diffraction pattern containing high-order Laue zone (HOLZ) reflections. (*a*) Schematic of reciprocal-lattice layers projected to form an electron diffraction pattern containing HOLZ reflections. (*b*) Calculation of the reciprocal-lattice layer spacing H^*^ by measuring the radius of the HOLZ ring *R*_H_. (*c*) Geometrical construction of a 3D primitive cell in reciprocal space. (*d*) Graphical user interface of the package *UnitCell Tools*.

Fig. 1 ▸(*a*) presents the geometric descriptions of a 3D reciprocal lattice in the TEM diffraction setting. The zone-axis electron diffraction pattern can contain both ZOLZ and HOLZ reflections as the Ewald sphere intersects with the reciprocal lattice of the crystal, where the ZOLZ pattern is only a 2D cross-section of the reciprocal lattice, but the HOLZ reflections can provide a wealth of 3D structural information.

### 2.1. The HOLZ ring and reciprocal-lattice layer spacing   

The formula and illustrations of the geometrical descriptions on the HOLZ ring and the reciprocal-layer spacing can be found in classic textbooks (De Graef, 2003 ▸; Williams & Carter, 2009 ▸). Here, we will describe in more detail how to derive this formula. Let us consider the layer spacing 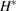 between the ZOLZ and FOLZ (first-order Laue zone) reciprocal layers along the incident beam direction [see Fig. 1 ▸(*b*)]. The layer spacing 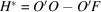, where *O*′*O* is the radius of the Ewald sphere, 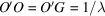 and λ is the wavelength of the incident electron beam. Assuming 2θ is the full scattering angle of the FOLZ ring, 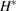 is rewritten as 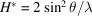 by considering 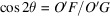 and 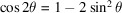. According to Bragg’s law 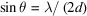, and assuming *R*_H_ = 1/*d* is the radius of the FOLZ ring, then 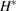 becomes
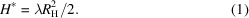
For other HOLZ rings with the order of the Laue zone *N*, the layer spacing becomes 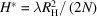. In practice, by measuring the radius of the HOLZ ring *R*_H_ (the units are nm^−1^ or Å^−1^), one can determine the reciprocal-lattice layer spacing 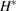 (the units are nm^−1^ or Å^−1^) along the incident beam direction.

### 2.2. Determination of unit-cell parameters from a single electron diffraction pattern   

More importantly, HOLZ reflections are essentially replicas of the ZOLZ ones, and therefore a full layer of HOLZ reflections can be generated by vector-addition of the basic vectors **OA** and **OB** which form the ZOLZ reflections, as shown in Fig. 1 ▸(*c*). Superimposing the full layer of FOLZ reflections onto the ZOLZ layer, one immediately recognizes that there must be a reflection *C* (in the FOLZ layer) that falls onto a position *C*_1_ within the 2D cell formed by vectors **OA** and **OB** in the ZOLZ layer.

The essence of the package *UnitCell Tools* is to establish a geometric relationship of a 3D reciprocal cell based on ZOLZ and HOLZ reflections [see Fig. 1 ▸(*c*)]. Since the line *CC*_1_ is perpendicular to the plane *AOB*, we write *CC*_1_⊥*OA*. If we draw a line segment *A*_1_ *C*_1_ perpendicular to the line *OA* passing through the projection spot *C*_1_, then the line *OA*_1_ is also perpendicular to the plane *CA*_1_ *C*_1_. Similarly, line *OB*_1_ is perpendicular to the plane *CB*_1_ *C*_1_ when we draw a line segment *B*_1_ *C*_1_ perpendicular to the line *OB*. One can easily construct a 3D reciprocal cell from *OA*, *OB*, *OA*_1_, *OB*_1_, *OC*_1_ and *CC*_1_, as expressed in Equation (2): (i) ZOLZ reflections define a 2D primitive cell by 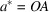, 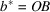 and 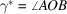; (ii) The HOLZ ring defines the reciprocal-layer spacing of *CC*_1_; (iii) The HOLZ diffraction spot provides the remaining parameters (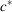, 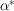 and 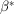) of a primitive cell.
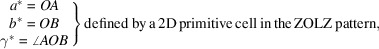


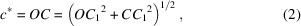
defined in the RtΔ*OC*_1_ *C*;
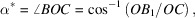
defined in the RtΔ*OB*_1_ *C*;
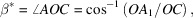
defined in the RtΔ*OA*_1_ *C*.

The constructed 3D reciprocal cell must be primitive if we choose a 2D primitive cell in the ZOLZ pattern and measure the innermost HOLZ ring. The constructed primitive cell can be further reduced to a Niggli cell, which can provide a unique description of a lattice (Křivý & Gruber, 1976 ▸; Guo, 1978 ▸; Grosse-Kunstleve *et al.*, 2004 ▸). In turn, the Niggli cell is uniquely converted to the unit cell of the Bravais lattice by employing the relationships well documented in Volume A of *International Tables for Crystallography: Space Group Symmetry* (Wolff, 2006 ▸).

### 2.3. Description of the software   

#### 2.3.1. Distribution and installation   

*UnitCell Tools* is a package of the *DigitalMicrograph* software (http://www.gatan.com/products/tem-analysis/gatan-microscopy-suite-software). The package can be provided via email upon reasonable request. The user should pre-install *DigitalMicrograph* software freely available at http://www.gatan.com/products/tem-analysis/gatan-microscopy-suite-software.

To install the package into the *DigitalMicrograph* software, copy the file ‘UnitCell Tools.gtk’ to ‘...\Gatan\DigitalMicrograph\PlugIns’, a new item ‘UnitCell Tools’ will be built on the menu bar. Click the item ‘UnitCell Tools/One Pattern’ to enter the graphical user interface, as shown in Fig. 1 ▸(*d*).

#### 2.3.2. Software overview   

*UnitCell Tools*, as a package of *DigitalMicrograph* software, can determine unit-cell parameters from electron diffraction patterns that contain ZOLZ reflections, HOLZ reflections and the HOLZ ring. It can process the native DM files (*.dm3) or other common greyscale images (JPG, TIF, BMP *etc*.). Before the pattern analysis, the diffraction pattern must be rigorously calibrated. The operation of the package is controlled via a graphical user interface [Fig. 1 ▸(*d*)], and processes for determining unit-cell parameters are described as follows:

(i) ZOLZ reflections are measured by successively locating four diffraction spots (by pressing the SPACE bar) around the transmitted spot after clicking the ‘Z’ button.

(ii) The HOLZ ring is measured by successively locating three points (by pressing the SPACE bar) on the HOLZ ring after clicking the ‘R’ button.

(iii) The HOLZ spot is measured (by pressing the SPACE bar) after clicking the ‘H’ button to construct a 3D primitive reciprocal cell. The three shortest vectors of the cell that meet the tolerant factors of ‘eps 1–3’ will be listed in the ‘Reduced Cell List’ box.

(iv) By choosing the appropriate basis vectors of the Niggli cell in the ‘Reduced Cell List’ box, the reduced cell can be converted into the unit cells. Unit cells that meet the limit of ‘eps d/A’ will be listed in the ‘Unit Cell List’ box.

(v) By picking the appropriate unit cell in the ‘Unit Cell List’ box, the simulated pattern based on the selected unit cell will be overlaid on the front pattern and the detailed parameters are output in the ‘Results’ box and the ‘Result window’.

Detailed operation of the package was documented in the user guide.

## 3. Results and discussion   

In this section, we will first illustrate how to extract unit-cell parameters from a simulated electron diffraction pattern based on the reported software, and then discuss some effects of experimental conditions (diffraction distortions, the misorientation of zone axis and the use of high-index zone axis) on the robustness of the package, and demonstrate the application of unit-cell determination of nanostructured materials. All experimental electron diffraction patterns were recorded on a JEM-2100 (Jeol Inc.) working at 200 kV.

### 3.1. Unit-cell determination from a simulated pattern   

**Figure 2:**
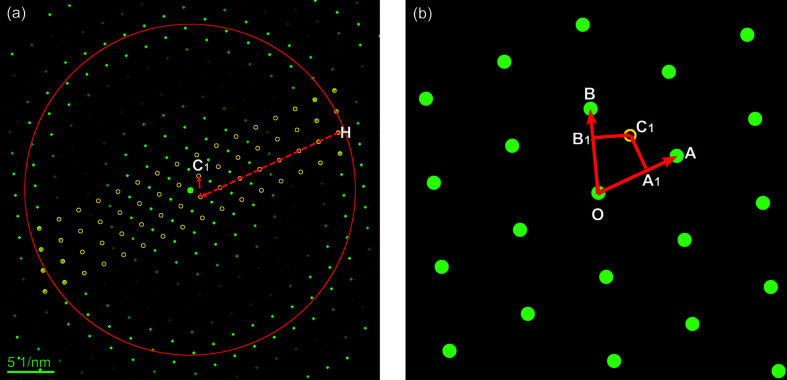
Illustrative construction of a 3D reciprocal cell using a simulated diffraction pattern with HOLZ reflections. (*a*) Simulated [211] zone-axis diffraction pattern with ZOLZ and HOLZ reflections for the monoclinic crystal La_2_(Ti_2_O_7_) (listed in Table 1 ▸). The red circle marks the HOLZ ring and the open yellow dots are generated by repeating the FOLZ reflections and then superimposed onto the ZOLZ layer. A typical FOLZ reflection, H, is shifted by integral multiples of the basic vectors of **OA** and **OB** to the position C_1_. (*b*) Enlarged ZOLZ pattern shows 2D geometrical relationship between the basic vectors **OA** and **OB**, and the spot *C*_1_ (shifted from H), together with the perpendicular intersection points *A*_1_ and *B*_1_.

Without loss of generality, we chose the monoclinic crystal structure of La_2_(Ti_2_O_7_) (the crystal structure is listed in Table 1 ▸). Fig. 2 ▸(*a*) presents a simulated [211] zone-axis electron diffraction pattern under the kinematic diffraction condition using the *EDA* software (Toshihiro, 2003 ▸), in which the first-order Laue zone (FOLZ) reflections and the ring marked by a red circle are clearly observed. The analysis procedure of unit-cell determination from a single electron diffraction pattern is as follows.

First, clicking the ‘Z’ button determines the 2D primitive cell with basic vectors of **OA** and **OB** in the ZOLZ layer [Fig. 2 ▸(*b*)] to be *a* ^*^ = *OA* = 0.2289 (2) Å^−1^, *b* ^*^ = *OB* = 0.2221 (1) Å^−1^ and *γ* ^*^ = *∠AOB* = 70.09 (1)°. For the dynamical diffraction pattern, we always suggest choosing the two shortest nonlinear reflections to form the 2D primitive cell; details are discussed in Section S1 of the supporting information.

Second, clicking the ‘R’ button determines the radius *R*_H_ of the FOLZ ring [red circle in Fig. 2 ▸(*a*)] to be 1.7330 (1) Å^−1^; according to Equation (1), *CC*_1_ = 0.0375 (1) Å^−1^. Note that, if the HOLZ ring is split, we always use the innermost ring (Williams & Carter, 2009 ▸). For convenience to measure the HOLZ ring by employing the three-point method, we suggest to acquire the HOLZ ring by selecting a large *C*_2_ aperture and focusing the beam. Details are discussed in Section S2 of the supporting information.

Third, clicking the ‘H’ button to locate a FOLZ reflection, H (2402.1, 939.1 pixels), which is then vector-shifted by the integral multiplier of **OA** and **OB** to *C*_1_ (1408.8, 1247.1 pixels), *i.e.* shift 7 units for **OA** and 1 unit for **OB**, as denoted in Fig. 2 ▸(*a*). By a simple geometric calculation [see Equation (2)], one can obtain a 3D reciprocal primitive cell with six parameters of *a* ^*^, *b* ^*^, *c* ^*^, *α* ^*^, *β* ^*^ and *γ* ^*^ (specific values are listed in Table 1 ▸).

Four, choosing three vectors in the ‘Reduced Cell List’ box to form the reduced cell, *e.g.* 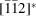, 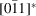 and 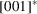. The obtained reciprocal reduced cell can then be converted to a real-space reduced cell via relationships among the real and reciprocal lattice parameters. The real-space Niggli cell is finally converted to the unit cell by applying the Niggli-to-Bravais cell transformation rule as tabulated in Volume A of *International Tables for Crystallography: Space Group Symmetry* (Wolff, 2006 ▸), and the cells which meet the limit of ‘eps d/A’ are listed in the ‘Unit Cell List’ box.

Five, picking the appropriate unit cell, *e.g.* the mP cell, the simulated pattern based on the selected unit cell (symmetry constraint cell) is overlaid on the front pattern and the detailed parameters are output in the ‘Results’ box and the ‘Output’ window. In the present case, the Niggli-matrix elements of the real-space Niggli cell are 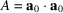 = 61.0273, 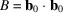 = 30.7419, 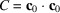 = 168.2796, 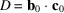 = −0.1869, 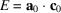 = −15.1522 and 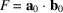 = −0.0222; the reduced Niggli cell meets the condition of *A* ≠ *B* ≠ *C*, *D* = *F* = 0 (No. 33, mP) and the Bravais lattice can be calculated by the corresponding matrix transformation to be *a* = 7.8120 (0), *b* = 5.5445 (5), *c* = 12.9723 (377) Å, α = 90.15 (15), β = 98.60 (6) and γ = 90.03 (3)°.

In order to estimate the accuracy of the cell, we separately evaluate the sum of the differences between the unit cell obtained and the standard Bravais as two figures of merit (FOM): one for unit-cell lengths of 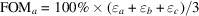 and the other for unit-cell angles of 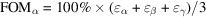, where 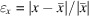 defines the absolute value of the relative error of unit-cell parameters. In order to correctly choose the appropriate unit cell and the reduced cell, the simulated pattern based on the selected unit cell is overlaid on the front pattern; parameters of the symmetry constraint cell and its variants, the zone-axis indices and the plane indices are listed in the ‘Results’ box. In the present case, we obtain the values of FOM_*a*_ = 0.10% and FOM_α_ = 0.09%; the zone axis is [211], and the plane indices of the reflections A and B are 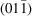 and 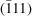, which are consistent with the simulated parameters.

It is worth noting that unit-cell parameters can be determined from a monoclinic crystal in this example, indicating the proposed package can be used in the case of low-symmetry crystal systems, *e.g.* the monoclinic and triclinic crystals. Instead, the tilt-series method is considered to be troublesome in the application for these two crystal systems (Li, 2019 ▸). As we will further demonstrate in real experimental data below, arbitrary zone-axis diffraction patterns, either low-symmetry or high-symmetry, can be used for unit-cell determination.

### 3.2. Effects of experimental conditions on the unit-cell determination   

#### 3.2.1. Diffraction distortions   

**Figure 3:**
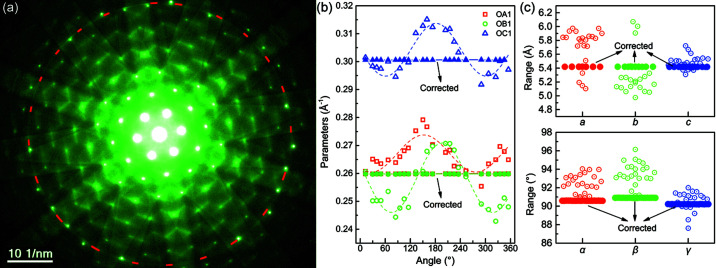
Effect of pattern distortions on unit-cell determination. (*a*) Typical 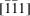-oriented diffraction pattern with HOLZ reflections of a silicon specimen. The red dashed circle marks the FOLZ ring used for measuring the reciprocal layer spacing H^*^. (*b*) Angle-dependent measured parameters *OA*_1_, *OB*_1_ and *OC*_1_ were extracted from 24 HOLZ reflections, and the dotted curves are the sine-function fitted curves. (*c*) Determined unit cells by using sets of angularly measured *OA*_1_, *OB*_1_ and *OC*_1_. Where the open symbols in Figs. 3 ▸(*b*)–3(*c*) represent the measured parameters of the uncorrected pattern, and the solid ones are those of the elliptical-distortion corrected pattern.

We now proceed to the unit-cell determination using experimental diffraction data. Fig. 3 ▸(*a*) shows a typical electron diffraction pattern of 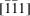-oriented silicon specimen recorded by a JEM-2100 microscope operated at 200 kV, in which a set of FOLZ reflections and a FOLZ ring marked by a red-dashed circle are clearly visible. Following the procedure detailed in the simulation case in the previous section, one can construct a 3D primitive reciprocal cell, the reciprocal Niggli cell, the real-space Niggli cell and the Bravais-lattice unit cell. In order to inspect the effects of diffraction distortions on the obtained unit-cell parameters, in the present case, using a set of FOLZ reflections around the FOLZ ring and the 2D primitive cell vectors **OA** and **OB**, one can obtain an angularly dependent set of *OA*_1_, *OB*_1_ and *OC*_1_ [Fig. 3 ▸(*b*)] and the unit cell of parameters *a*, *b*, *c*, α, β and γ [Fig. 3 ▸(*c*)]. The open symbols represent the parameters of the uncorrected diffraction pattern, and the solid ones represent those of the elliptical-distortion corrected pattern. Such angular variations in the 3D unit-cell determination are actually found to exist, as the experimental diffraction pattern invariably bears diffraction distortions (Mugnaioli *et al.*, 2009 ▸). The angularly fitted sine-function curves in Fig. 3 ▸(*b*) also indicate that the dominant distortion in the electron diffraction pattern is elliptical, mainly due to twofold aberrations of the projection lens and/or objective lens – higher angle scattered electrons (*e.g.* HOLZ reflections) traveling close to the polepieces of the respective lens cause image distortions. Consequently, distortions of the diffraction pattern cause measurement errors in (i) the measurement of the HOLZ ring, which may cause an unreliable spacing of the reciprocal layer *CC*_1_ (it has a minor effect on the determined cell, details are discussed in Section S2 of the supporting information); (ii) measurement of the HOLZ diffraction spot, which may lead to inaccurate values of *OA*_1_, *OB*_1_ and *OC*_1_, in turn, affecting the determination of reciprocal primitive cell parameters *c* ^*^, α^*^ and β^*^. So, a high-resolution diffraction pattern with well defined HOLZ reflections suffering minimal image distortions is essential to determine unit-cell parameters.

Despite the presence of diffraction distortions, similar unit-cell parameters [see open dots in Fig. 3 ▸(*c*)] are obtained, illustrating the robustness of the unit-cell determination by employing single electron diffraction that contains HOLZ reflections. However, it is advised to accurately correct diffraction distortions prior to performing the unit-cell determination, a more accurate unit cell can be determined using the corresponding HOLZ reflection after distortion correction [see the solid dots in Fig. 3 ▸(*c*)].

#### 3.2.2. The misoriented zone-axis pattern   

**Figure 4:**
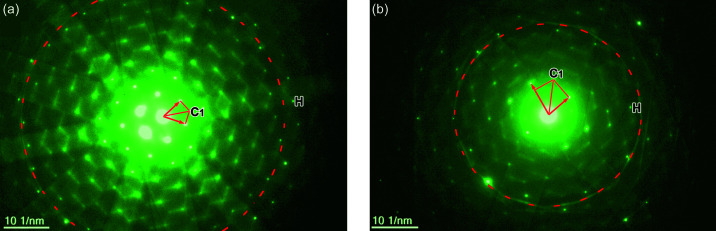
Effect of diffraction conditions on the unit-cell determination: (*a*) an electron diffraction pattern of silicon deviated from the exact zone-axis 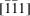 condition by about 0.37°, and (*b*) a low-symmetry and high-index 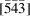 zone axis pattern of silicon.

We address the effect of electron diffraction patterns with slight misorientation of the zone axis on the robustness and accuracy of unit-cell determination. In general, exactly oriented zone-axis diffraction patterns are ideal for accurate unit-cell determination. Such conditions are not always obtainable as they are either limited by the tilting accuracy of specimen holders or the difficulty in precise tilting of nanosized specimens (Zhao *et al.*, 2008 ▸; Li, 2019 ▸; Zou *et al.*, 2004 ▸). Here, to inspect the effects of misorientation of zone axis on unit-cell determination, we deliberately misoriented the Si specimen by ∼0.37° from its exact 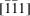 zone axis, as shown in Fig. 4 ▸(*a*). Using this diffraction pattern, we carried out the procedure for unit-cell determination as detailed in the previous section and tabulated the measured values of the 3D reciprocal primitive cell, the reciprocal Niggli cell, the real-space Niggli cell and the Bravais-lattice unit cell in Table 1 ▸. When comparing the unit cell determined with the known lattice parameters of silicon, the low FOMs (FOM_*a*_ = 0.01% and FOM_α_ = 0.44%) indicate that the proposed software works well under even misoriented zone-axis conditions. It should be noted that the intensities of ZOLZ reflections will be asymmetric when the incident beam deviates from the zone axis. In this case, we suggest measuring high-index reflections (*Nh* *Nk* *Nl*) around the transmitted spot to reduce measurement errors of *OA* and *OB* resulting from the deviation vector.

#### 3.2.3. High-index and low-symmetry patterns   

We next address the effect of using high-index and low-symmetry zone-axis electron diffraction patterns on unit-cell determination. Conventional methods for unit-cell determination require the use of major zone-axis diffraction patterns, namely those with low index and higher symmetry (Zou *et al.*, 2004 ▸; Li, 2019 ▸; Zhao *et al.*, 2008 ▸). By contrast, the package also works well on arbitrary zone-axis diffraction patterns as long as the patterns contain HOLZ reflections. In fact, in some cases, the use of a high-index and lower symmetry pattern is advantageous as HOLZ reflections and rings are close to the ZOLZ ones with higher visibility for easy and accurate measurements.

To illustrate the effect, we recorded a high-index 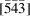 zone axis pattern of the Si specimen [Fig. 4 ▸(*b*)] and then performed the unit-cell determination procedure. The resulting values are tabulated in Table 1 ▸. The low values of FOM (FOM_*a*_ = 0.55% and FOM_α_ = 0.31%) verify that the package can accurately determine unit-cell parameters from high-index and low-symmetry zone axis diffraction patterns.

#### 3.2.4. Determination of unit-cell parameters of small crystallites   

The merit of the package can be best illustrated by the determination of unit-cell parameters of small crystallites. As compared with X-ray and neutron scattering methods of the broad-beam nature, electron diffraction in a TEM is a superior means for probing microstructures of finite-sized specimens, such as nanoparticles, nanoscale inclusions or precipitates (Shi, Zou *et al.*, 2019 ▸; Antion *et al.*, 2003 ▸). However, the small-sized crystallites are difficult to tilt to multiple zone-axis conditions, which greatly limits the use of the conventional reciprocal-cell reconstruction method in the case of small crystallites.

**Figure 5:**
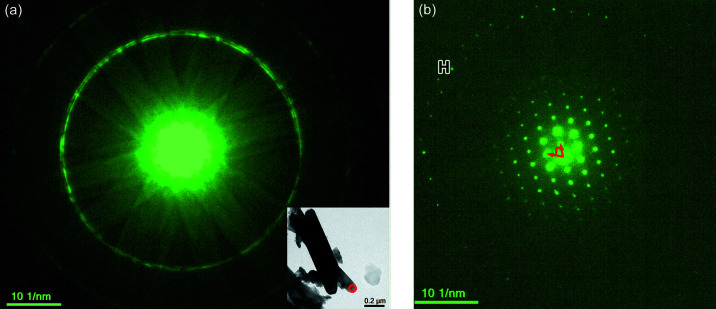
Determination of unit-cell parameters from a nano-rod TiO_2_ oriented at 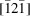: (*a*) convergent-beam mode of TEM can provide a sharp HOLZ ring, (*b*) nano-beam mode of TEM can capture the electron diffraction from a small nano-crystallite.

We demonstrate the applicability of the package for determining unit-cell parameters of TiO_2_ nanorods. Fig. 5 ▸(*a*) shows a convergent-beam electron diffraction pattern of TiO_2_ nanorods, in which FOLZ and SOLZ (second-order Laue zone) rings are clearly visible. The radius of the FOLZ ring is used for determining the reciprocal-lattice layer spacing H^*^ along the electron beam direction. By keeping the diffraction condition but using a smaller condensed lens aperture (∼10 µm in diameter), nanobeam electron diffraction is produced as shown in Fig. 5 ▸(*b*), in which discrete ZOLZ and FOLZ reflections are clearly seen. Following the procedure from constructing a 3D reciprocal primitive cell to the Bravais lattice as detailed in the previous section, we obtained a set of unit-cell values that are displayed in Table 1 ▸. The low FOM values (FOM_*a*_ = 0.67% and FOM_α_ = 0.90%) compared the experimentally determined unit cell with the known orthorhombic phase of TiO_2_ (PDF#76–1937, *a* = 5.472, *b* = 5.171, *c* = 9.211 Å, α = β = γ = 90°) indicate the examined TiO_2_ nanorods are of the brookite type. However, we note that FOM values are relatively higher, likely due to the larger measurement errors in the diffraction pattern, *e.g.* the poor-defined and unsharp reflections, and the low signal-to-noise ratio of the pattern.

#### 3.2.5. Remarks on the recording of high-quality electron diffraction patterns   

The above examples indicate that unit-cell parameters can be determined from a single electron diffraction pattern by employing the proposed software, although the accuracy of the determined parameters remains difficult to compare with those of the X-ray method due to the unreliable camera constant and the aberrations present in the TEM (Mugnaioli *et al.*, 2009 ▸). Here, we give some general remarks on the recording of high-quality electron diffraction patterns with well defined ZOLZ and HOLZ reflections, accurately calibrated camera length, and minimal image distortions as follows:

(1) Prior to the electron diffraction pattern acquisition, one should perform standard microscope optical alignment procedures, including alignment of the illumination system, centering of the voltage/current, centering of the condenser-lens/selected-area aperture, correcting the condenser-/diffraction-lens astigmatism and adjustment of the sample eucentric height. An optimized electron optic is beneficial to obtain a sharp and well defined diffraction pattern.

(2) Calibration of the camera length with a standard specimen to set the correct image scale.

(3) Lowering the accelerating voltage of TEM can enhance the HOLZ reflections and increase the view of the pattern with the same camera length.

(4) Appropriate choice of camera length and exposure time for recording electron diffraction patterns with clear HOLZ reflections. In some cases, a double-exposure method can be applied: short exposure time to obtain ZOLZ spots and long exposure time for HOLZ reflections.

(5) Lowering the temperature of the specimen can reduce the thermo-diffusion scattering and hence enhance the HOLZ reflections.

(6) Mitigation of the imaging distortions from the intermediate and projection lenses by adjustment of their lens aberrations and misalignment using a clean diffraction pattern from standard specimens.

## 4. Conclusions   

We developed an analysis package to determine unit-cell parameters of crystals using a single electron diffraction pattern that contains both ZOLZ and HOLZ reflections. The proposed software was verified by a simulated electron diffraction pattern together with detailed working procedures. We then carried out experiments using diffraction data of the silicon specimen to evaluate the effects of diffraction distortions, the misorientation of the zone axis, the use of high-index and lower symmetry zone-axis patterns on the robustness and accuracy of the unit-cell determination. Moreover, we experimentally demonstrated the applicability of the proposed package for the unit-cell determination of TiO_2_ nanorods. In this particular case, it is advised to use convergent-beam electron diffraction to extract the radius of the FOLZ ring and nano-beam electron diffraction to locate the ZOLZ reflections and one of the HOLZ reflections.

Compared with other reciprocal-space reconstruction methods (Zhao *et al.*, 2008 ▸; Zou *et al.*, 2004 ▸; Li, 2019 ▸), our proposed method requires only one pattern that needs to not be in the exact zone-axis condition and needs to not be a low-index zone axis, this will greatly simplify the TEM operation. The accuracy of the determined parameters is slightly better than the series tilt method (details are discussed in Section S3 of the supporting information) but are still not comparable with those of X-ray methods; the parameters determined can be regarded as the first step and can be then refined by X-ray or neutron diffraction. For complex materials of multiphases or small sizes (typically, 50–100 nm), this is probably the sole choice for the structural identification and determination of new crystal structures.

## Supplementary Material

Demonstration for the package UnitCell Tools. DOI: 10.1107/S2052252521007867/gq5014sup1.wmv

The reported software. DOI: 10.1107/S2052252521007867/gq5014sup2.bin

The tested pattern. DOI: 10.1107/S2052252521007867/gq5014sup3.bin

Supporting figures and tables. DOI: 10.1107/S2052252521007867/gq5014sup4.pdf

## Figures and Tables

**Table 1:** Examples of unit-cell determination The row ‘Parameters’ includes the parameters *OA*_1_, *OB*_1_, *OC*_1_, *CC*_1_, *A*_1_ *C*_1_ and *B*_1_ *C*_1_, respectively. The units are Å^−1^ for the reciprocal space, Å for the real space and ° for the angles.

(1) Simulated pattern of crystal La_2_(Ti_2_O_7_) with a low crystal system (FOM_*a*_ = 0.10% and FOM_α_ = 0.09%): PDF#81-1066, *P*2_1_, *a* = 7.812, *b* = 5.544, *c* = 13.010 Å, α = 90.00, β = 98.66, γ = 90.00°, oriented at [211].						
Parameters	0.1422 (0)	0.1466 (1)	0.1764 (0)	0.0375 (1)	0.1044 (1)	0.0982 (0)
Reciprocal cell	0.2289 (2)	0.2221 (1)	0.1804 (0)	35.65 (2)	37.96 (2)	70.09 (1)
Reduced cell	0.0780 (2)	0.1295 (0)	0.1804 (0)	89.95 (5)	89.85 (15)	81.40 (6)
Direct cell	7.8120 (0)	5.5445 (5)	12.9723 (377)	90.15 (15)	98.60 (6)	90.03 (3)
Unit cell	7.8120 (0)	5.5445 (5)	12.9723 (377)	90.15 (15)	98.60 (6)	90.03 (3)
						
(2) SAED pattern deviated by ∼0.37° from the exact zone axis [{\bar 1\bar 11}] of silicon specimen (FOM_*a*_ = 0.01% and FOM_α_ = 0.44%). The standard cell is PDF#77-2108, *a* = *b* = *c* = 5.42 Å, α = β = γ = 90.00°.						
Parameters	0.5254 (35)	0.5250 (31)	0.6048 (22)	0.1077 (12)	0.2996 (17)	0.3002 (11)
Reciprocal cell	0.5234 (15)	0.5227 (8)	0.6141 (22)	31.25 (23)	31.19 (29)	59.46 (54)
Reduced cell	0.3180 (16)	0.3186 (10)	0.3199 (3)	109.39 (8)	109.32 (15)	109.14 (33)
Direct cell	3.8265 (60)	3.8217 (108)	3.8099 (226)	60.39 (39)	60.43 (43)	60.54 (54)
Unit cell	5.4202 (2)	5.4193 (7)	5.4205 (5)	90.32 (32)	90.68 (68)	90.18 (18)
						
(3) High-index and low-symmetry pattern oriented at [{\bar 5\bar 4\bar 3}] of the silicon specimen (FOM_a_ = 0.55% and FOM_α_ = 0.31%). The standard cell is PDF#77-2108, *a* = *b* = *c* = 5.42 Å, α = β = γ = 90.00°.						
Parameters	0.6017 (96)	0.6290 (53)	0.7955 (62)	0.0521 (0)	0.5204 (17)	0.4870 (34)
Reciprocal cell	0.6055 (58)	0.8047 (13)	0.7972 (62)	37.91 (5)	41.00 (54)	78.61 (59)
Reduced cell	0.3182 (11)	0.3183 (10)	0.3198 (5)	110.12 (65)	109.32 (15)	108.58 (89)
Direct cell	3.8155 (208)	3.8331 (32)	3.8319 (44)	59.85 (15)	60.35(35	60.79 (79)
Unit cell	5.4196 (57)	5.3837 (416)	5.4727 (474)	90.16 (16)	90.57 (57)	90.12 (12)
						
(4) TiO_2_ nano-rod oriented at [\bar{1}2 \bar{1}] (FOM_*a*_ = 0.67% and FOM_α_ = 0.90%). The standard cell is PDF#76-1937, *a* = 5.472, *b* = 5.171, *c* = 9.211 Å, α = β = γ = 90.00°.						
Parameters	0.1684 (2)	0.1320 (19)	0.1823 (4)	0.0643 (14)	0.0699 (18)	0.1257 (13)
Reciprocal cell	0.2209 (9)	0.2890 (16)	0.1934(0)	46.94 (76)	29.46 (15)	66.14 (17)
Reduced cell	0.1086 (0)	0.1793 (34)	0.1934(0)	89.08 (92)	89.42 (58)	89.02 (98)
Direct cell	*a*_0_ = 5.5792 (1072)	*b*_0_ = 5.1718 (8)	*c*_0_ = 9.2066 (44)	α_0_ = 90.56 (56)	β_0_ = 90.97 (97)	γ_0_ = 90.91 (91)
Unit cell	*a* = 5.5792 (1072)	*b* = 5.1718 (8)	*c* = 9.2066 (44)	α = 90.56 (56)	β = 90.97 (97)	γ = 90.91 (91)

## References

[bb1] Altomare, A., Cuocci, C., Moliterni, A. & Rizzi, R. (2019). *International Tables for Crystallography*, Volume H, edited by C. J. Gilmore, J. A. Kaduk & H. Schenk. Chichester: John Wiley.

[bb2] Antion, C., Donnadieu, P., Perrard, F., Deschamps, A., Tassin, C. & Pisch, A. (2003). *Acta Mater.* **51**, 5335–5348.

[bb3] Ayer, R. (1989). *J. Elec. Microsc. Tech.* **13**, 16–26. 10.1002/jemt.1060130105 2674366

[bb4] Baer, D. R., Amonette, J. E., Engelhard, M. H., Gaspar, D. J., Karakoti, A. S., Kuchibhatla, S., Nachimuthu, P., Nurmi, J. T., Qiang, Y., Sarathy, V., Seal, S., Sharma, A., Tratnyek, P. G. & Wang, C.-M. (2008). *Surf. Interface Anal.* **40**, 529–537.

[bb5] Capitani, G. C., Oleynikov, P., Hovmöller, S. & Mellini, M. (2006). *Ultramicroscopy*, **106**, 66–74. 10.1016/j.ultramic.2005.06.003 16046067

[bb6] Collier, C. P., Vossmeyer, T. & Heath, J. R. (1998). *Annu. Rev. Phys. Chem.* **49**, 371–404. 10.1146/annurev.physchem.49.1.371 15012432

[bb7] David, W. I. F., Shankland, K., Mccusker, L. B. & Baelocher, C. (2006). *Structure Determination from Powder Diffraction Data*. Oxford University Press. 10.1107/S0108767307064252 18156673

[bb8] De Graef, M. (2003). *Introduction to Conventional Transmission Electron Microscopy*. Cambridge University Press.

[bb10] Grosse-Kunstleve, R. W., Sauter, N. K. & Adams, P. D. (2004). *Acta Cryst.* A**60**, 1–6. 10.1107/s010876730302186x 14691322

[bb11] Guo, K. X. (1978). *Acta Phys. Sin.* **27**, 160–168.

[bb12] Hou, V. D. H. & Li, D. (2008). *Microscopy Today*, **16**, 36–41.

[bb13] Křivý, I. & Gruber, B. (1976). *Acta Cryst.* A**32**, 297–298.

[bb14] Lábár, J. L. (2008). *Microsc. Microanal.* **14**, 287–295.

[bb15] Le Bail, A., Duroy, H. & Fourquet, J. L. (1988). *Mater. Res. Bull.* **23**, 447–452.

[bb16] Li, X. Z. (2019). *Micron*, **117**, 1–7. 10.1016/j.micron.2018.10.010 30408701

[bb17] Mugnaioli, E., Capitani, G., Nieto, F. & Mellini, M. (2009). *Am. Mineral.* **94**, 793–800.

[bb18] Pecharsky, V. K. & Zavalij, P. Y. (2003). *Fundamentals of Powder Diffraction and Structural Characterization of Materials*. Boston, MA: Springer US.

[bb19] Shi, H. L., Luo, M. T. & Wang, W. Z. (2019). *Comput. Phys. Commun.* **243**, 166–173.

[bb20] Shi, H. L., Zou, B., Li, Z. A., Luo, M. T. & Wang, W. Z. (2019). *Beilstein J. Nanotechnol.* **10**, 1434–1442. 10.3762/bjnano.10.141 PMC6664412 31431855

[bb21] Toshihiro, K. (2003). *Jpn Mag.f Mineral. Petrol. Sci.* **32**, 96–101.

[bb22] Williams, D. B. & Carter, C. B. (2009). *Transmission Electron Microscopy: a Textbook for Materials Science*. Boston, MA: Springer US.

[bb23] Wolff, P. M. de (2006). *International Tables for Crystallography.* Vol. A. ch. 9.2, pp. 750–755. International Union of Crystallography.

[bb24] Young, R. A. (1995). *The Rietveld Method*. Oxford University Press.

[bb25] Zhao, H. S., Wu, D. Q., Yao, J. C. & Chang, A. M. (2008). *Ultramicroscopy*, **108**, 1540–1545. 10.1016/j.ultramic.2008.05.001 18555610

[bb26] Zou, X. D., Hovmöller, A. & Hovmöller, S. (2004). *Ultramicroscopy*, **98**, 187–193. 10.1016/j.ultramic.2003.08.025 15046798

[bb27] Zuo, J. M. (1992). *Ultramicroscopy*, **41**, 211–223.

[bb28] Zuo, J. M. (1993). *Ultramicroscopy*, **52**, 459–464.

[bb29] Zuo, J. M., Lábár, J. L., Zhang, J., Gorelik, T. E. & Kolb, U. (2018). *International Tables for Crystallography*, pp. 102–117. International Union of Crystallography.

