# Supplementary material for: UnitCell Tools, a package to determine unit-cell parameters from a single electron diffraction pattern
Source: IUCrJ. 2021 Aug 20;8(Pt 5):805–13. doi: 10.1107/S2052252521007867 (PMC8420764; doi:10.1107/S2052252521007867)
Supplement: Supplementary file 4 [file m-08-00805-sup4.pdf]

IUCrJ

Volume 8 (2021)

Supporting information for article:

UnitCell Tools, a package to determine unit-cell parameters from a single electron diffraction pattern

Hong-Long Shi and Zi-An Li

S1. Comparison of the unit cell determination between kinematical and dynamical conditions

To demonstrate the validation of the proposed method in the kinematical and the dynamical conditions, we acquired a dynamical diffraction pattern, as shown in Figure S2. In this pattern, it contains the kinematical reflections and the dynamical reflections (indicated by yellow arrows). Unit cell parameters were determined from both kinematical and dynamical conditions, i.e., 2D primitive cells were chosen as OA and OB for the Kinematical case and OA_1 and OB for the dynamical case. The detailed parameters were listed in Table S1. It indicated that both kinematical and dynamical reflections can be used to determine unit cell parameters but with the different types of the Bravais lattice (e.g., tI and tP, respectively). Hence, we suggest that one always chooses the two shortest nonlinear reflections as the 2D primitive cell.

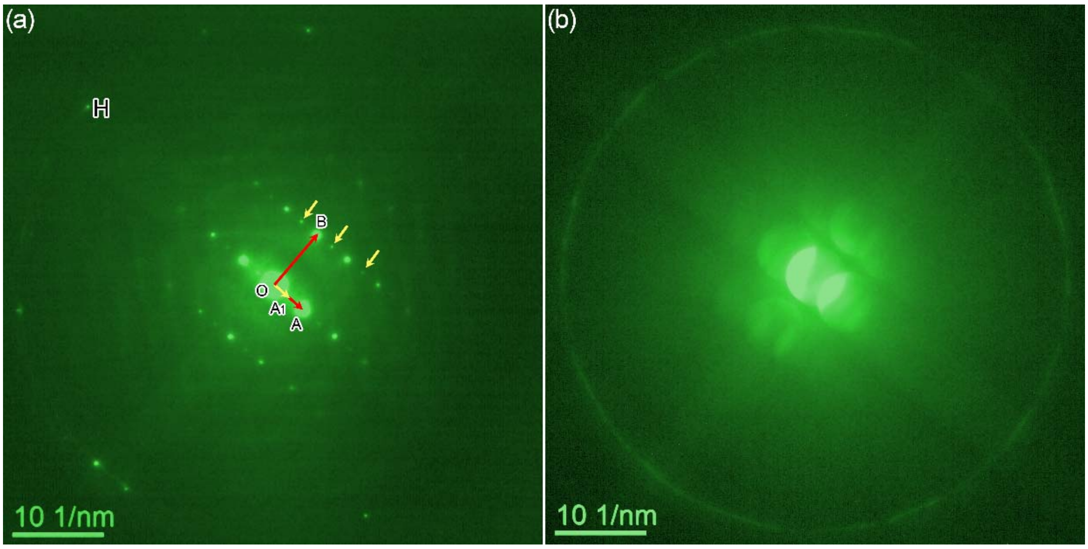

Figure S1 Unit cell determination from a dynamical electron diffraction pattern of the rutile crystallite, where reflections pointed by yellow arrows are dynamical ones. 2D primitive cells were chosen as OA and OB for the kinematical condition and OA_1 and OB for the dynamical case.

Table S1 Comparison of the cell parameters determined from kinematical and dynamical reflections of a rutile crystallite (PDF#75-1755, P 4_2/m n m, a=b=4.594 Å, c=2.959 Å, α=β=γ=90°).

Cell type	Kinematical condition, tI(7)	Dynamical condition, tP(11)
Reciprocal cell	4.3006, 7.2755, 3.0734, 49.81, 45.24, 89.47	2.1503, 7.2755, 3.0496, 49.94, 45.19, 89.47
Reduced cell	3.0539, 3.8211, 3.8529, 47.22, 66.59, 67.13	2.1637, 2.1503, 3.2043, 91.27, 90.73, 90.03
Direct cell	3.6250, 3.6227, 3.6072, 128.37, 101.37, 100.16	4.6221, 4.6517, 3.1218, 88.73, 89.27, 89.95
Unit cell	4.5825, 4.6508, 3.1486, 89.54, 89.97, 89.63	4.6221, 4.6517, 3.1218, 88.73, 89.27, 89.95

S2. Effect of the radius of the HOLZ ring on the unit cell parameters

To inspect the effect of the radius of the HOLZ ring on the unit cell parameters based on the proposed method, we change the radius of the HOLZ ring of Figure 3 from 514.936 pixels to 509.936 and 519.936 pixels, respectively. The determined cells were listed in Table S2. It indicated that the obtained unit cells possess similar parameters though the radius of the HOLZ ring deviates 5 pixels from the value determined by the three-point method, suggesting the radius of the HOLZ ring has a minor effect on the unit cell parameters.

Table S2 Effect of the radius of the HOLZ ring on the unit cell parameters.

HOLZ ring	R_H=509.936 pixels or 29.1372 nm^{-1}, H^*=1.0646 nm^{-1}			R_H=514.936 pixels or 29.4229 nm^{-1}, H^*=1.0856 nm^{-1}			R_H=519.936 pixels or 29.7086 nm^{-1}, H^*=1.1068 nm^{-1}		
Reciprocal cell	5.2348	5.2275	6.1440	5.2348	5.2275	6.1477	5.2348	5.2275	6.1515
	31.24	31.18	59.46	31.30	31.24	59.46	31.36	31.30	59.46
Reduced cell	3.1812	3.1868	3.1938	3.1883	3.1939	3.2225	3.1955	3.2012	3.2297
	109.41	109.35	109.12	109.34	109.26	108.76	108.98	108.89	108.40
Direct cell	3.8262	3.8208	3.8178	3.7959	3.7912	3.7687	3.7483	3.7436	3.7213
	60.37	60.40	60.55	60.71	60.77	61.07	61.57	61.62	61.93
Unit cell	5.4260	5.4164	5.4251	5.3905	5.3811	5.3916	5.3575	5.3473	5.3587
	90.37	90.56	90.21	90.53	91.30	90.37	91.25	92.02	91.10

S3 Comparison between the proposed method and the series tilt method

For comparison, we determine unit cell parameters by the series tilt method. It took more than half an hour to strictly orient the silicon crystal to two zone-axis directions, as shown in Figure S1(a-b). The tilt angle between two zone axes is derived to be 7.95°. The reconstructed reciprocal cell is a^*=0.5097 Å, b^*=0.5062 Å, c^*=0.7882 Å, α^*=13.09°, β^*=70.87°, γ^*=59.93°; the direct cell is a=3.764 Å, b=3.858 Å, c=3.942 Å, α=63.12°, β=59.58°, γ=64.83°; and the unit cell is a=5.7884 Å, b=5.3073 Å, c=5.5648 Å, α=89.60°, β=92.06°, γ=94.68°. The FOM_a and FOM_α are 3.85% and 2.64%, worse than those of the proposed method (e.g., 0.55% and 0.31% for the $[\bar{5}\bar{4}\bar{3}]$ of silicon specimen).

The major problem of the series tilt method is the misfit of the scalar factors of patterns because the camera constant of each pattern may change even by a few percent in the same microscope and in the same session(Mugnaioli *et al.*, 2009). Instead, the proposed method needs only one pattern to determine unit cell parameters and so without consideration of the misfit problem as that in the series tilt method.

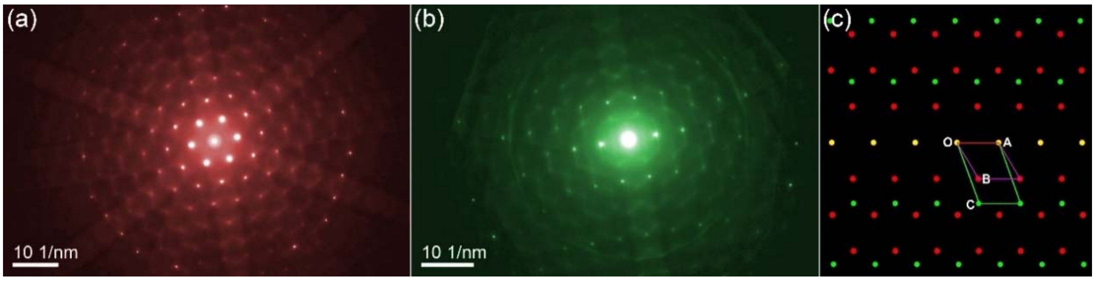

Figure S2 Unit cell determination based on the series tilt method: (a-b) two experimental SAED patterns, and (c) the overlaid pattern, where the red spots are extracted from (a), the green spots from (b), and the yellow spots are the common diffraction spots.

Mugnaioli, E., Capitani, G., Nieto, F. & Mellini, M. (2009). *American Mineralogist* **94**, 793-800.
